# Preparation of Amidoxime Polyacrylonitrile Chelating Nanofibers and Their Application for Adsorption of Metal Ions

**DOI:** 10.3390/ma6030969

**Published:** 2013-03-11

**Authors:** Fenglin Huang, Yunfei Xu, Shiqin Liao, Dawei Yang, You-Lo Hsieh, Qufu Wei

**Affiliations:** 1 Key Laboratory of Eco-Textiles, Ministry of Education, Jiangnan University, Wuxi 214122, China; E-Mails: flhuang@jiangnan.edu.cn (F.H.); yunfeixu1990@126.com (Y.X.); Shiqin_Liao@yahoo.cn (S.L.); yangdawei891@gmail.com (D.Y.); 2 University of California Davis, Davis, CA 95616, USA; E-Mail: ylhsieh@ucdavis.edu

**Keywords:** nanofibers, polyacrylonitrile, amidoxime polyacrylonitrile, adsorption

## Abstract

Polyacrylonitrile (PAN) nanofibers were prepared by electrospinning and they were modified with hydroxylamine to synthesize amidoxime polyacrylonitrile (AOPAN) chelating nanofibers, which were applied to adsorb copper and iron ions. The conversion of the nitrile group in PAN was calculated by the gravimetric method. The structure and surface morphology of the AOPAN nanofiber were characterized by a Fourier transform infrared spectrometer (FT-IR) and a scanning electron microscope (SEM), respectively. The adsorption abilities of Cu^2+^ and Fe^3+^ ions onto the AOPAN nanofiber mats were evaluated. FT-IR spectra showed nitrile groups in the PAN were partly converted into amidoxime groups. SEM examination demonstrated that there were no serious cracks or sign of degradation on the surface of the PAN nanofibers after chemical modification. The adsorption capacities of both copper and iron ions onto the AOPAN nanofiber mats were higher than those into the raw PAN nanofiber mats. The adsorption data of Cu^2+^ and Fe^3+^ ions fitted particularly well with the Langmuir isotherm. The maximal adsorption capacities of Cu^2+^ and Fe^3+^ ions were 215.18 and 221.37 mg/g, respectively.

## 1. Introduction

With the rapid development of global industry and the advent of new technologies, environmental contamination has presented a great threat to human health; especially with significantly the amounts of heavy metal ions in wastewater [1]. Heavy metal pollution is characterized by its strong concealing ability and accumulative damage, and it cannot be biodegraded. Heavy metals are not biodegradable, thus hazardous to the ecological environment and, more seriously, can cause various diseases, e.g., headaches, nausea, vomiting, abdominal pain, insomnia, forgetfulness, neurological disorders and liver damage [2,3,4]. Thus, the removal and recovery of heavy metals has become one of the most predominant aspects of environment research.

Various techniques have been utilized to remove and recycle heavy metals from aqueous solutions such as chemical precipitation, ion exchange, membrane separation, electrochemical treatment, adsorption, *etc.* [5,6,7,8]. Among them, adsorption is one of the most simple and common methods. The adsorption of metal ions can be achieved by using polymer materials containing specific functional groups, for example, amino, carboxyl, phosphoric, tetrazine and amidoxime, *etc.* [1], to form strong complexes with metal ions via the coordination reaction. The adsorption of metals onto these materials mainly depends on the functional groups on the adsorbent surfaces. Amidoxime group, in particular, has exhibited superior adsorption ability because it contains both amino and carboxyl groups.

Recently, the electrospinning technique, a simple and versatile method, has been widely applied to produce nanofibers. Electrospun nanofibers are known to possess numerous interesting characteristics such as high porosity, small interfibrous pore size, and most importantly much largee specific surface in comparison to conventional fibers. Nanofibers are applied widely in tissue engineering [9], drug delivery [10], sensors [11], protective clothing [12], fine filtration and adsorptive membranes [13,14]. The high specific surface makes nanofibers better adsorbents [15], which have high adsorption rates and capacities than other types of materials such as resins, foams, and conventional fibers, *etc.* [16]. Thus, nanofibers, modified by introducing functional groups on their surface, could be applicable for the removal and recovery of heavy metals from aqueous solutions.

Polyacrylonitrile (PAN) has been recognized as a highly efficient material for the removal and enrichment of heavy metals [1]. In the present study, PAN nanofibers were prepared by the electrospinning technique and further chemically modified with hydroxylamine to synthesize chelating nanofibers. The conversion of nitrile group in the PAN molecule was calculated gravimetrically. The structure and surface morphology of amidoxime PAN (AOPAN) nanofibers were analyzed by a Fourier transform infrared spectrometry (FT-IR) and a scanning electron microscopy (SEM), respectively. The modified nanofibers were subsequently applied to adsorb copper and iron ions from aqueous solutions. The concentrations of copper and iron ions were measured by atomic absorption spectroscopy (AAS). The adsorption isotherm was constructed at a given temperature of 303.15 K. The equilibrium parameters were also calculated.

## 2. Experimental Section

### 2.1. Materials

The PAN powders (*M*w = 30,000–50,000 g/mol) were purchased from America. The ferric chloride (FeCl_3_·6H_2_O), copper chloride (CuCl_2_·2H_2_O), and *N*,*N*-Dimethyl Formamide (DMF) were obtained from SCRC. All the chemicals were analytical grade and were used without further purification.

### 2.2. Instrumentation

The surface morphologies of raw PAN and AOPAN nanofibers were analyzed using an SEM, Quanta-200 from HITACHI (Japan). A Fourier transform infrared (FT-IR) spectrometer, Nicolet is10, from Thermo Fisher Scientific Inc. (China) was employed to analyze the chemical structure. A Spectr AA-220 atomic absorption spectroscopy (AAS) was used to measure the concentration of copper and iron ions in solutions.

### 2.3. Electrospinning of PAN Nanofibers

The PAN powders were dissolved in DMF at 10 wt % concentration under constant stirring at room temperature for 24 h to make even spinning solution. The prepared solution was loaded in a 10 mL of plastics syringe with a metal needle (0.3 mm inner diameter and 0.7 mm external diameter). The electrospinning parameters were determined based on the previous research achievements [17]. The applied electrical voltage, distance between the needle tip and collector, and flow rate were fixed at 18 kV, 15 cm and 0.5 mL/h, respectively. Under the high voltage, the droplet was ejected, accelerated towards the collector in an external electrostatic field and collected on the surface of aluminum foil as a nanofibrous mat.

### 2.4. Chemical Modification of PAN Nanofibers

**Scheme 1:**
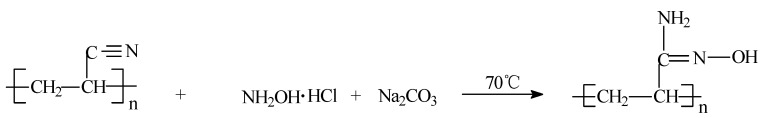
The reaction between hydroxylamine and nitrile group.

Electrospun PAN nanofibers were modified into chelating nanofibers containing amidoxime groups by reacting with hydroxylamine hydrochloride, transforming nitrile groups into amidoxime groups as shown in Scheme 1. Dried samples of PAN nanofiber mats were immersed in a 50 mL of aqueous solution of hydroxylamine hydrochloride for some time. The pH value was adjusted with anhydrous sodium carbonate. After reaction, the nanofibrous membranes were washed with deionized water several times and then dried in an oven at 323 K.

The conversion of nitrile group in the PAN was calculated as follows [18]:

(1) $$C=\frac{W_{1}{\text{-W}}_{\text{0}}}{{\text{W}}_{\text{0}}}\times \frac{53}{33}\times 100$$

where *C* (%) is the conversion of nitrile group into amidoxime group in the PAN, *W*_0_ is the weight of the initial PAN nanofiber membranes, *W*_1_ is the weight of the modified PAN nanofiber membranes, 53 and 33 are the molecular weights of acrylonitrile monomer and hydroxylamine, respectively.

### 2.5. Adsorption Behaviors

All adsorption experiments were conducted in 250 mL of beakers at 303 K. Dried raw PAN nanofiber and modified nanofiber mats, with the same total specific surface area, were immersed in 50 mL aqueous metal salt solutions for different time duration, and then removed from the beakers at 0.5, 1, 2, 3, 6, 24 h. The obtained nanofiber mats were washed with deionized water. The lotion and the remaining metal salt aqueous solutions were transferred to a 100 mL of volumetric flask, evenly mixed for measurement. The equilibrium isotherm time was determined, and the equilibrium isotherm was investigated at 72 h and at 303 K. The concentration of metal-ions in solutions was measured by atomic absorption spectroscopy (AAS). The adsorption amounts were calculated as follows:

(2) $$Q\left(\text{mg}/g\right)=\frac{C_{0}V_{0}-C_{1}V_{1}}{m}$$

where *Q* is the adsorption amount (mg/g) onto the PAN nanofibers, *C*_0_ is the initial concentration of metal ions (mg/L), *C*_1_ is the final concentration (mg/L), * m* is the weight of nanofiber mats (g).

## 3. Results and Discussion

### 3.1. FT-IR Study

**Figure 1:**
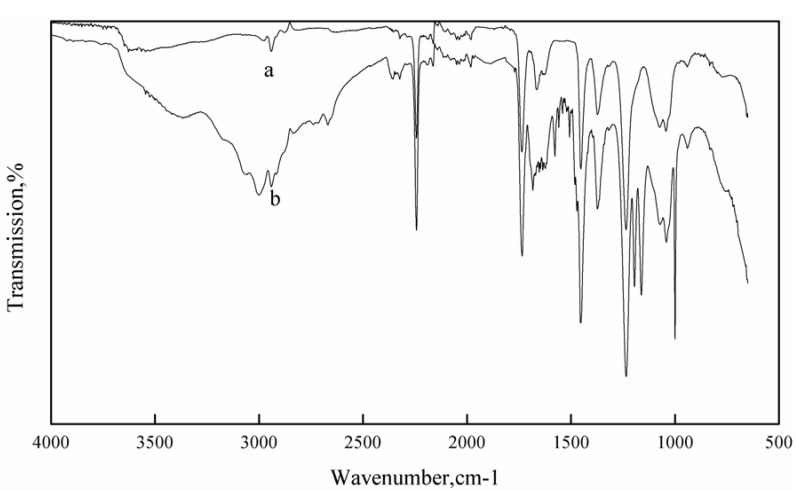
Fourier transform infrared spectrometer (FT-IR) spectra of raw polyacrylonitrile (PAN) and amidoxime polyacrylonitrile (AOPAN) nanofiber mats (**a**) Raw PAN; (**b**) Modified PAN.

In this study, the FT-IR spectrometer was utilized to investigate the chemical reaction between hydroxylamine and the nitrile group on PAN. The FT-IR spectra of raw PAN and AOPAN nanofiber mats are shown in Figure 1. The FT-IR spectrum of raw PAN (Figure 1a) presented the characteristic absorption peak at 2243 cm^−1^ (–C≡N) and 1735 cm^−1^ (C=O), which indicated that the PAN was a copolymer or the DMF solvents and not volatilized completely. The FT-IR spectrum of modified PAN (Figure 1b) exhibited correlative characteristic bands of amidoxime at 3100 cm^−1^, 1577 cm^−1^, 1506 cm^−1^, 1161 cm^−1^, and 1000 cm^−1^, which was attributed to the stretching vibration of O–H, C=N, N–H, C–N and N–O, respectively [19]. The FT-IR spectra ratified that the amidoxime group was introduced onto the PAN surface. The peak at 2243 cm^−1^ in the FT-IR spectrum of modified PAN suggested that nitrile groups in PAN were partly converted into amidoxime groups.

### 3.2. Conversion of Nitrile Group

Table 1 shows the effects of the reaction conditions on the conversion of nitrile group in PAN and the appearance properties of modified PAN nanofibers.

**Table 1:** Effects of the reaction conditions on conversion and appearance properties.

NO.	Time (h)	Temperature (K)	NH_2_OH content (g/L)	pH value	Conversion (%)	Appearance and properties
1	1	313.15	40	7	21.3	Soft and white
2	2	313.15	40	7	26.4	Soft and white
3	3	313.15	40	7	43.6	Brittle and white
4	4	313.15	40	7	51.7	Brittle and light yellow
5	5	313.15	40	7	56.2	Brittle and light yellow
6	2	333.15	40	3	26.0	Soft and white
7	2	333.15	40	5	50.6	Brittle and light yellow
8	2	333.15	40	7	57.4	Brittle and light yellow
9	2	333.15	40	9	31.4	Soft and white
10	2	333.15	40	11	22.2	Soft and white
11	2	343.15	10	7	27.8	Soft and white
12	2	343.15	20	7	44.1	Soft and white
13	2	343.15	30	7	59.3	Brittle and light yellow
14	2	343.15	40	7	78.1	Hard and light yellow
15	2	343.15	50	7	83.8	Hard and light yellow
16	2	313.15	40	7	26.4	Soft and white
17	2	323.15	40	7	32.8	Soft and white
18	2	333.15	40	7	58.4	Brittle and light yellow
19	2	343.15	40	7	78.1	Hard and light yellow
20	2	353.15	40	7	86.0	Hard and light yellow

As shown in Table 1, the conversion of the nitrile group in the PAN molecules increased along with the increase of reaction time, reaction temperature and concentration of hydrochloride hydroxylamine. Maximum conversion was achieved at pH 7. This could be attributed to the various chemical specifications of hydroxylamine in different pH conditions. NH_2_OH·HCl existed in the acid condition, which reduced the amount of free hydroxylamine. Although the alkaline condition promoted the generation of free hydroxylamine, the molecule would be unstable and volatile. The conversion depended on the amount of hydroxylamine molecule diffused from the reaction solution into the PAN nanofibers. The increasing reaction temperature and hydroxylamine concentration effectively promoted the molecular diffusion of hydroxylamine into the PAN nanofibers. Longer reaction times also improved the molecular diffusion of hydroxylamine from the solution into the nanofibers and increased the reaction probability between hydroxylamine and the nitrile groups. Hydrochloride hydroxylamine in the solution predominantly exited in the form of free hydroxyl amine molecules at pH 7, which accelerated the conversion of nitrile group.

The color of the PAN nanofiber mats changed from white to light yellow and the mats became brittle or hard with increasing conversion of nitrile group in the PAN. These color changes probably occurred because of the long heating time in reaction process [1]. The decrease of the softness may be due to the high conversion of nitrile group into amidoxime group, because the larger molecules contributed more significantly to strength and toughness compared to the shorter molecules [18].

### 3.3. SEM

**Figure 2:**
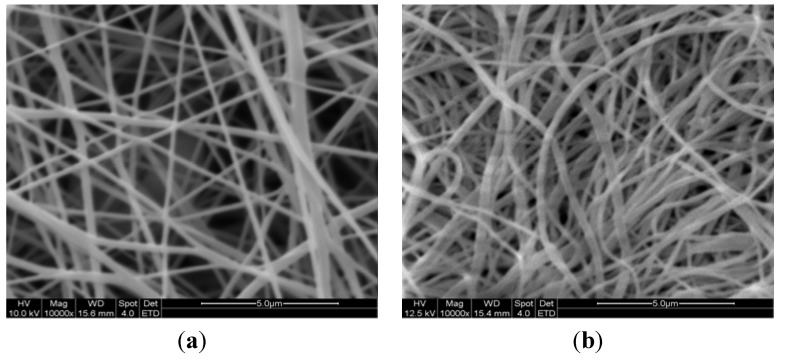
Scanning electron microscope (SEM) photos of (**a**) PAN nanofibers; (**b**) AOPAN nanofibers (53% conversion).

The SEM photos of the raw PAN nanofiber and AOPAN nanofiber are shown in Figure 2. The morphologies were similar to each other, and the surface of AOPAN nanofiber did not show any serious cracks or sign of degradation. Figure 2b shows how the modified PAN nanofibers became expanded and bent compared to the raw PAN nanofibers. In the electrospinning process, the electrical force at the surface of the drop solution overcame the solution surface tension, and then the polymer solution was stretched and elongated into nanofibers. Consequently the polymers in the PAN nanofibers were in the high-energy metastable state. In the wet heating process, the polymers relaxed to a lower energy state, which was attributed to the swelling and contracting of nanofibers.

### 3.4. Adsorption Behaviors

**Figure 3:**
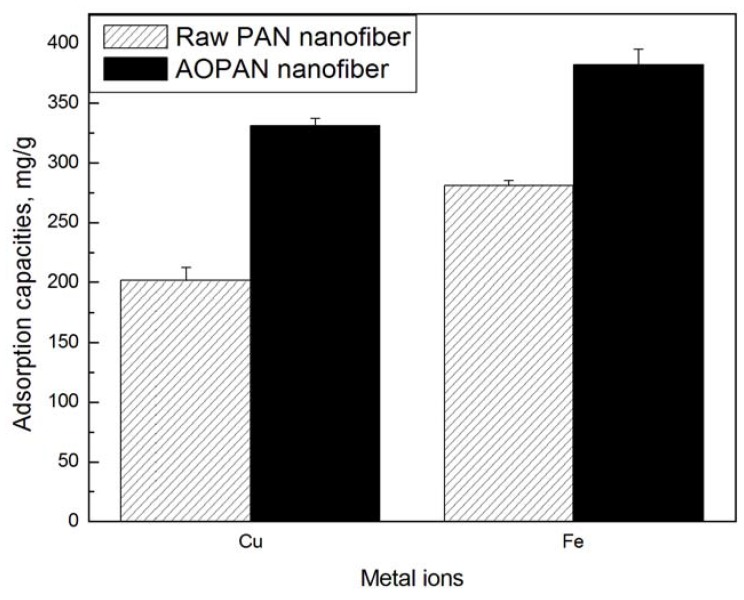
Adsorption capacities of copper and iron ions onto the PAN and AOPAN nanofibers.

Figure 3 shows the adsorption amount of Cu^2+^ and Fe^3+^ ions onto the PAN nanofiber mats and AOPAN nanofiber mats (57.4% conversion) in a 1 mg/L of solution as a function of time (for 72 h). The experiment for each sample was repeated five times. The error analysis was also shown in the figure. It was evident that the chemical modification of PAN nanofibers had a significant effect on the adsorption ability. The adsorption capacities of Cu^2+^ and Fe^3+^ ions onto PAN nanofibers were 198.46 and 278 mg/g, respectively. However, the capacities onto AOPAN nanofibers were 320 and 380 mg/g, respectively. The capacities adsorbed on the PAN nanofibers were obviously lower than those onto the AOPAN nanofibers. The results in Figure 3 confirmed that the introduction of amidoxime group on the AOPAN indeed strengthened the adsorption ability of metal ions.

It was observed that the adsorption capacity of Fe^3+^ ion onto nanofibers was higher than that of Cu^2+^ ion, as shown in Figure 3. The difference of adsorption capacity of metal ions depended on their special affinities with the active adsorption sites on PAN nanofibers at the same initial concentration. Fe^3+^ ion is a hard acid, with a small size and strong electropositive, which more easily accepted electrons from the ligand. However, Cu^2+^ ion displayed a weaker electron-accepting nature compared to Fe^3+^ ion. PAN and AOPAN, containing hydroxyl and amine group, had the structure characteristics of hard acid, with an electron-donating nature and strongly electronegativity. According to HSAB (Hard-Soft Acid Base) theory and Lewis acid-base theory [20], Fe^3+^ ions were expected to give stronger complexes with PAN and AOPAN, which led to Fe^3+^ ion having a higher adsorption capacity than Cu^2+^ ion.

**Figure 4:**
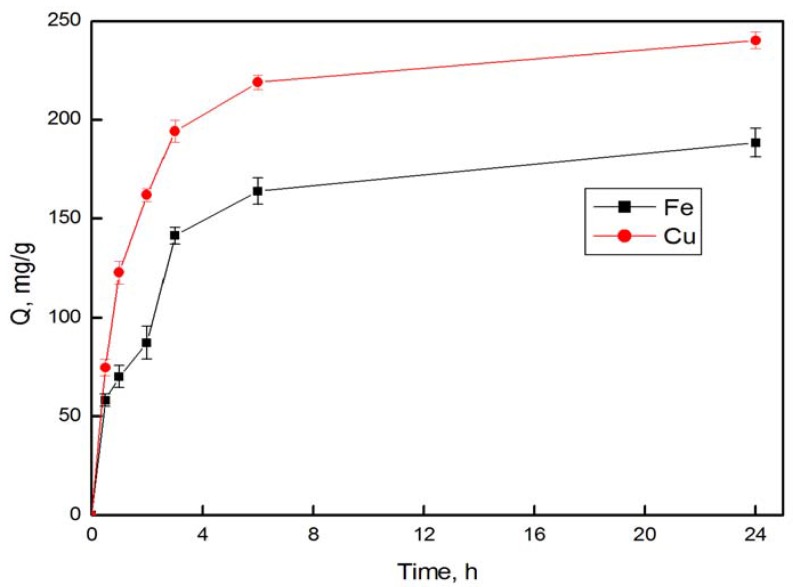
Adsorption of Cu^2+^ and Fe^3+^ ions on the AOPAN (43.6% conversion) nanofiber mat, in a 500 ppm solution as a function of time.

Figure 4 illustrates the adsorption of copper and iron ions into the AOPAN nanofiber mats (43.6% conversion) as a function of time (24 h). The amount of adsorption increased rapidly until 3 h and then leveled off. The rapid increase in the initial 3 h was due to the abundant available chelating oxime sites on the surface of AOPAN nanofibers and the high concentration of metal ions [21]. The adsorption rates decreased and finally reached equilibrium because of the depletion of the adsorptive sites as well as the decrease of metal-ion concentrations in the solution. According to Figure 4, the adsorption time of 72 h was determined to study the adsorption equilibrium amounts of copper and iron ions.

**Figure 5:**
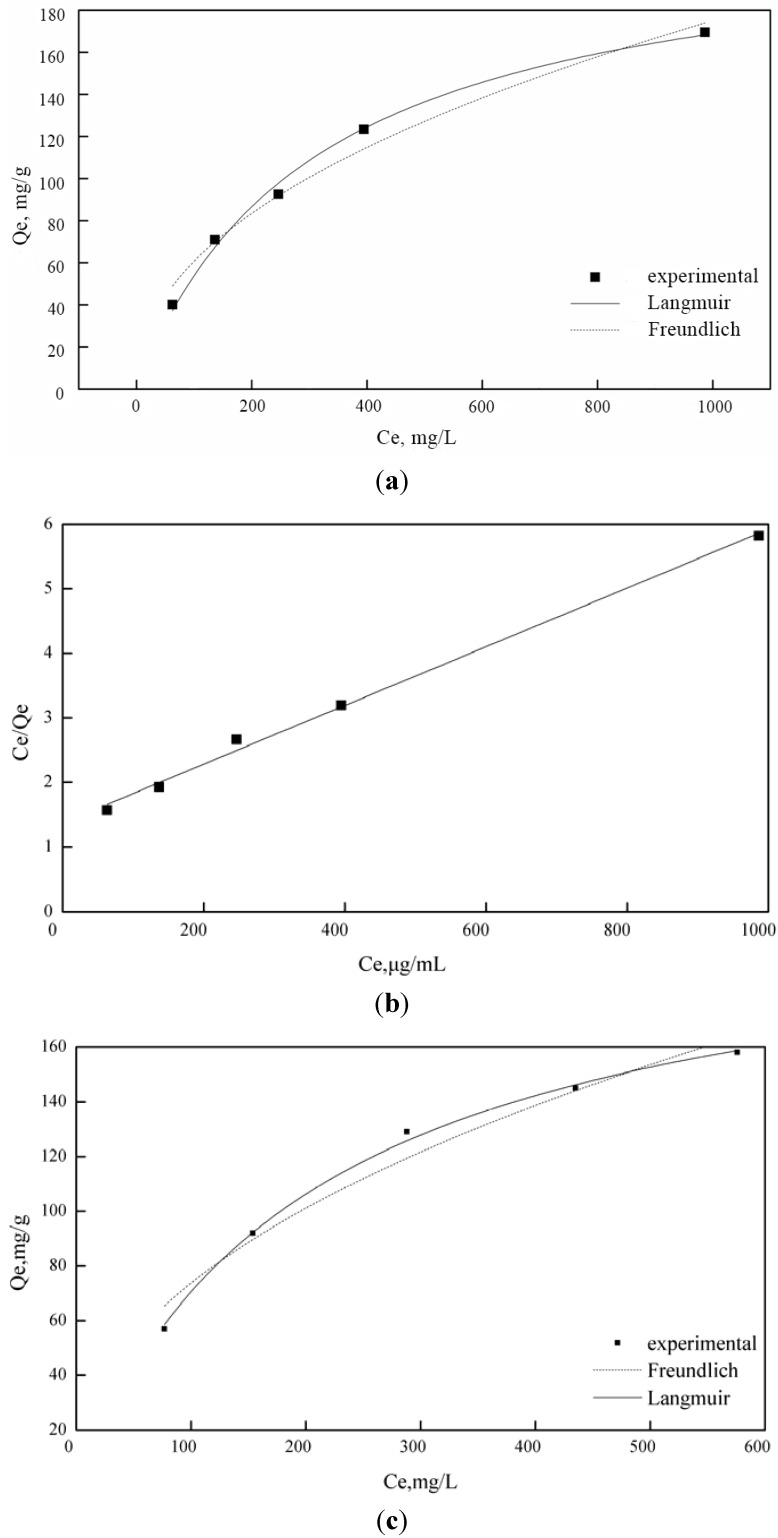

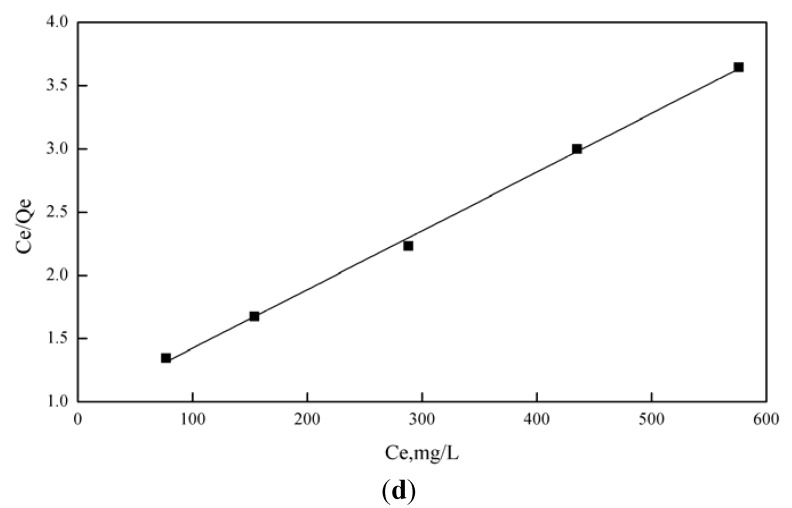
Adsorption isotherms of iron ions on the (**a**) AOPAN (31.4% conversion) fitting models; (**b**) Langmuir model at 303 K and adsorption isotherms of copper ions on the (**c**) AOPAN (36% conversion) fitting models; (**d**) Langmuir model at 303 K.

Figure 5 illustrates the equilibrium adsorption amounts (*Q*_e_) of Cu^2+^ and Fe^3+^ ions onto AOPAN nanofiber mats after the equilibrium time (72 h) as a function of equilibrium concentration (*C*_e_). *Q*_e_ increased sharply and then gradually with an increase in *C*_e_. At equilibrium, an adsorption isotherm can be constructed at a given constant temperature. Adsorption data should accurately fit into different isotherm models. The most common ones are the Langmuir and the Freundlich models. These equations are as follows, respectively:

(3) $$Q_{\text{e}}=\frac{Q_{\text{m}}bC_{\text{e}}}{1+bC_{\text{e}}}$$

where *Q*_e_ is the equilibrium amount of the metal adsorbed onto the AOPAN nanofiber mat (mg/g); *Q*_m_ is the maximum adsorption capacity (mg/g); *b* is the Langmuir constant related to binging energy (L/mg); *C*_e_ is the equilibrium concentration (mg/L). The values of *Q*_m_ and *b* were calculated, as shown in Table 2.

(4) Qe=KCe1n

where *K* and *n* are the Freundlich constants. The values of these parameters were also analyzed from the plots shown in Figure 5, given in Table 2.

**Table 2:** Langmuir and Freundlich constants for metal-ion adsorption on AOPAN nanofiber mats.

Metal ions	Langmuir model			Freundlich model		
	*Q*_m_ (mg/g)	*b* (L/mg)	*r*^2^	*K* (mg^(1−1/n)^ L^1/n^ g^−1^)	*n*	*r*^2^
Cu^2+^	215.17829	0.00488	0.99693	9.03894	0.45573	0.96072
Fe^3+^	221.3681	0.00321	0.99337	7.29263	0.46002	0.97463

As shown in Table 2, the experimental adsorption data of copper and iron ions onto the AOPAN nanofiber mats fitted better to the Langmuir model than the Freundlich model, as indicated by the very high values of the correlation coefficient (*r*^2^). The Langmuir model is used to describe the adsorption taking place at specific homogeneous sites. Once an adsorptive site is occupied, no further adsorption can occur. Thus, the adsorption of copper and iron ions followed the formation of a monolayer. The reported value for the maximum adsorption of Cu^2+^ ion onto AOPAN nanofibers is approximately that of Fe^3+^ ion. However, this value is not consistent with the results shown in Figure 3 because the conversions of nitrile group in AOPAN were significantly different in this study.

## 4. Conclusions

The AOPAN nanofiber mats were produced using the electrospinning technique and chemical modification of the nitrile group. The conversion of the nitrile group in PAN increased along with increased reaction time, temperature and concentrations of hydrochloride hydroxylamine. The conversion was maximized at pH 7. The PAN nanofiber mats became light yellow and brittle above 50% conversion. SEM photos confirm that the AOPAN nanofiber did not show any serious cracks or degradation. The adsorption capacities of copper and iron ions onto the AOPAN nanofiber mats were higher than the PAN nanofiber mats. The adsorption of both Cu^2+^ and Fe^3+^ ions on the AOPAN nanofiber mat increased with increase in adsorption time, and then leveled off at approximately 6 h. The adsorption data of both Cu^2+^ and Fe^3+^ ions fitted particularly well with the Langmuir isotherm, indicating that adsorption took place via the formation of a monolayer.

## References

[1] Neghlani P.K., Rafizadeh M., Taromi F.A. (2011). Preparation of aminated-polyacrylonitrile nanofiber membranes for the adsorption of metal ions: Comparison with microfibers. J. Hazard. Mater..

[2] Jain C.K., Singhal D.C., Sharma M.K. (2004). Adsorption of zinc on bed sediment of River Hindon: Adsorption models and kinetics. J. Hazard. Mater..

[3] Sekar M., Sakthi V., Rengaraj S. (2004). Kinetics equilibrium adsorption study of lead(II) onto activated carbon prepared from coconut shell. J. Colloid Interface Sci..

[4] Iqbal M., Saeed A., Zafar S.I. (2007). Hybrid biosorbent: an innovative matrix to enhance the biosorption of Cd(II) from aqueous solution. J. Hazard. Mater..

[5] Miretzky P., Saralegui A., Cirelli A.F. (2006). Simultaneous heavy metal removal mechanism by dead macrophytes. Chemosphere.

[6] Shoushtari A.M., Zargaran M., Abdouss M. (2006). Preparation and characterization of high efficiency ion-exchange crosslinked acrylic fibers. J. Appl. Polym. Sci..

[7] Shin D.H., Ko Y.G., Choi U.S., Kim W.N. (2004). Design of high efficiency chelate fibers with an amine group to remove heavy metal ions and pH-related FT-IR analysis. J. Ind. Eng. Chem. Res..

[8] Tahaei P., Abdouss M., Edrissi M., Shoushtari A.M., Zargaran M. (2008). Preparation of chelating fibrous polymer by different diamines and study on their physical and chemical properties. Mater. Sci. Eng. Technol..

[9] Pham Q.P., Sharma U., Mikos A.G. (2006). Electrospinning of polymeric nanofibers for tissue engineering applications: A review. J. Tissue Eng..

[10] Kenawy E.R., Bowlin G.L., Mansfield K. (2002). Release of tetracycline hydrochloride from electrospun poly(ethylene-*co*-vinylacetate), poly(lactic acid), and a blend. J. Control. Release.

[11] Liu H., Kameoka J., Czaplewski D.A., Craighead H.G. (2004). Polymeric nanowire chemical sensor. J. Nano. Lett..

[12] Gibson P., Schreuder-Gibson H., Rivin D. (2001). Transport properties of porous membranes based on electrospun nanofibers. J. Colloid Surf. A Physicochem. Eng. Asp..

[13] Sang Y., Gu Q., Sun T. (2008). Filtration by a novel nanofiber membrane and alumina adsorption to remove copper (II) from groundwater. J. Hazard. Mater..

[14] Sang Y., Li F., Gu Q., Liang C.Z., Chen J.Q. (2008). Heavy metal-contaminated groundwater treatment by a novel nanofiber membrane. J. Desalin..

[15] Ma Z.W., Masaya K., Ramakrishna S. (2006). Immobilization of Cibacron blue F3GA on electrospun polysulphone ultra-fine fiber surfaces towards developing an affinity membrane for albumin adsorption. J. Membr. Sci..

[16] Saeed K., Haider S., Oh T.J., Park S.Y. (2008). Preparation of amidoxime-modified polyacrylonitrile (PAN-oxime) nanofibres and their applications to metal ions adsorption. J. Membr. Sci..

[17] Cai Y.B., Gao D.W., Wei Q.F., Gu H.L., Huang F.L., Song L. (2011). Effects of ferric chloride on structure, surface morphology and combustion property of electrospun polyacrylonitrile composite nanofibers. Fibers Polym..

[18] Lin W.P., Lu Y., Zeng H. (1993). Studies of the preparation, structure, and properties of an acrylic chelating fiber containing amidoxime groups. J. Appl. Polym. Sci..

[19] Bilba N., Bilba D., Moroi G. (2004). Synthesis of a polyacrylamidoxime chelating fiber and its efficiency in the retention of palladium ions. J. Appl. Polym. Sci..

[20] Coskun R., Soykan C. (2009). Preparation of amidoximated polyester fiber and competitive adsorption of some heavy metal ions from aqueous solution onto this fiber. J. Appl. Polym. Sci..

[21] Deng S.B., Bai R., Chen J.P. (2003). Aminated polyacrylonitrile fibers for lead and copper removal. Langmuir.

